# A Novel Methodological Approach to Measure Linear Trends in Health Inequalities: Proof of Concept With Adolescent Smoking in Europe

**DOI:** 10.1093/aje/kwad029

**Published:** 2023-02-03

**Authors:** Mirte A G Kuipers, Kaidi Kang, Anca D Dragomir, Karin Monshouwer, Elisa Benedetti, Gabriele Lombardi, George Luta, Anton E Kunst

**Keywords:** adolescents, eradication of inequalities, Europe, inequality, simulation methods, socioeconomic status

## Abstract

We describe a new method for presenting and interpreting linear trends in health inequalities, and present a proof-of-concept analysis of inequalities in smoking among adolescents in Europe. We estimated the regression line of the assumed linear relationship between smoking prevalence in low– and high–socioeconomic status (SES) youth over time. Using simulation, we constructed a 95% confidence interval (CI) for the smoking prevalence in low-SES youth for when this would be 0% in high-SES youth, and we calculated the likelihood of eradicating smoking inequality (<5% for both low and high SES). This method was applied to data on adolescents aged 15–16 years (*n* = 250,326) from 23 European countries, derived from the 2003–2015 European Survey Project on Alcohol and Other Drugs. Smoking prevalence decreased more slowly among low- than among high-SES adolescents. The estimated smoking prevalence was 9.4% (95% CI: 6.1, 12.7) for boys and 5.4% (95% CI: 1.4, 9.2) for girls with low SES when 0% with high SES. The likelihood of eradicating smoking inequality was <1% for boys and 37% for girls. We conclude that this novel methodological approach to trends in health inequalities is feasible in practice. Applying it to trends in smoking inequalities among adolescents in Europe, we found that Europe is currently not on track to eradicate youth smoking across SES groups.

## Abbreviations

CI: confidence interval
CR: confidence region
ESPAD: European Survey Project on Alcohol and Other Drugs
SES: socioeconomic status

Governments increasingly aim to reduce health inequalities by implementing policies that improve health equity (1–3). To monitor the impact of such policies, epidemiologists need accurate measurements of trends in inequalities. A key decision in describing health inequalities is whether to measure absolute or relative inequalities, that is, the difference or the ratio of health outcome rates between groups of interest (e.g., socioeconomic groups, ethnic groups) (4). According to a 2012 literature review, inequalities were most commonly reported using only relative measures (5). The authors concluded that this decision may strongly influence the conclusions we draw about trends or variations in health inequalities (5).

More recently, public health researchers increasingly present both absolute and relative measures of inequality (6–12). Although this is more transparent than presenting only absolute or relative measures, this approach may lead to ambivalent conclusions (4, 7, 13, 14). For example, Moser et al. (14) demonstrated that a ranking of countries on their magnitude of health inequalities depended on whether the rate ratio or rate difference was used, and that trends in absolute and relative inequality measures had opposite directions in over a fifth of the countries. The latter is particularly problematic as this complicates monitoring whether countries are on the right track to reach their goals. There is also concern that methods of measuring trends are selected to best fit the narrative (15). It would therefore be beneficial to develop a method to present trends in health inequalities in a way that allows drawing a singular, unambiguous conclusion from given data, thereby moving beyond a choice between absolute and relative perspectives.

The presentation of trends in health inequalities and the conclusions drawn from it should focus not only on the magnitude of inequalities but also on the extent to which the trend heads in the desired direction, if the current trend were to continue. For outcomes that are declining in their prevalence, desirability may be expressed as the extent to which prevalence will reach values below 5% across population subgroups, a scenario of “eradication of inequalities.” This would allow trends in inequalities for declining outcomes to be judged for how much they agree with this desirable scenario.

This study aimed to develop an integrated method to present and interpret linear trends in health inequalities. The proposed method does not require the explicit measurement of either absolute or relative differences. Instead, it offers a quantification of “likelihood of eradication,” which is more meaningful when striving for equality in eradication. To provide a proof of concept, we used data on adolescent smoking in 23 European countries from 4 cross-sectional surveys (2003–2015). Previous studies established that although the prevalence of smoking among young people in Europe is decreasing, there are substantial socioeconomic inequalities (13, 16–20) that may even increase over time (21). In “Europe’s Beating Cancer Plan” of 2021, the European Union has set the goal of reducing smoking prevalence to below 5% before 2040 (22). Similar to the 2017 World Health Organization’s report on “Tobacco-Free Generations” (23), the EU’s plan acknowledges the importance of preventing smoking among youth and investing specifically in prevention among groups with lower socioeconomic status (SES). Adolescent smoking is therefore an example of a health-related outcome that would benefit from an integrated assessment of trends in socioeconomic inequalities.

The specific objectives of this study were:

- To develop a graphical representation of linear trends in health inequalities across SES groups
- To develop a method to determine the likelihood of an eradication scenario (<5% prevalence in both high- and low-SES groups) if trends in inequalities were to continue
- To provide a proof of concept by applying these methods to smoking prevalence among adolescents in 23 European countries, across 4 European regions

## METHODS

### Empirical data

#### Country selection.

In this paper we utilized data from the European Survey Project of Alcohol and Other Drugs (ESPAD) surveys. ESPAD collects quadrennial survey data of repeated cross-sectional samples of 15- to 16-year-old adolescents across Europe. In total, 27 countries collected data for all 4 survey waves. Three countries were excluded because their data did not include SES measurement in all years (Cyprus, Germany, and Portugal), and 1 country was excluded because the total sample size was smaller than 4,000 (Faroe Islands). As a result, data from 23 countries were included in this study; see Table 1.

**Table 1:** Smoking Prevalence in 23 European Countries, for Boys and Girls and According to Region and Country, European Survey Project on Alcohol and Other Drugs Over 4 Survey Waves, 2003–2015^a^

	**Boys**						**Girls**					
**Region and Country**		**Smoking Prevalence, %**						**Smoking Prevalence, %**				
	**Total No.**	**2003–2015**	**2003**	**2007**	**2011**	**2015**	**Total No.**	**2003–2015**	**2003**	**2007**	**2011**	**2015**
All regions and countries	120,823	22.2	26.6	22.7	22.7	16.1	129,503	21.4	25.0	22.4	21.6	16.2
North	35,849	19.6	24.9	21.1	19.2	11.6	37,928	17.6	22.1	19.3	17.5	10.5
Denmark	2,760	17.9	20.8	23.5	19.3	9.5	3,154	17.9	21.9	25.1	14.5	13.2
Estonia	4,451	24.1	33.6	25.5	20.3	16.3	4,539	19.7	26.2	17.6	21.7	12.8
Finland	6,199	22.2	27.2	22.6	23.8	16.1	6,915	22.2	30.1	22.1	24.1	13.9
Iceland	5,691	9.1	14.7	11.3	6.0	3.0	5,694	10.0	14.5	13.6	6.9	3.9
Latvia	3,742	33.7	35.4	35.7	36.5	18.5	4,030	25.9	24.8	28.8	29.8	13.6
Lithuania	4,236	30.9	43.4	29.6	28.7	19.7	4,517	19.8	21.4	20.2	23.0	14.1
Norway	4,372	9.0	14.3	9.7	6.3	3.5	4,361	12.2	22.2	12.9	5.9	3.5
Sweden	4,398	9.8	9.6	10.6	12.6	6.0	4,718	13.4	15.4	14.4	14.0	9.0
East	40,438	26.8	32.3	25.9	27.0	20.8	45,029	25.5	28.0	26.0	26.2	21.2
Bulgaria	4,498	29.5	35.6	30.2	26.6	25.7	4,714	36.6	41.9	37.6	36.7	30.5
Czech Republic	6,233	30.8	36.6	30.0	34.2	20.2	7,058	34.6	37.1	38.5	35.8	24.6
Hungary	5,644	26.8	30.7	25.8	28.1	21.5	5,550	26.2	30.1	27.1	25.6	21.4
Poland	10,265	19.5	26.4	16.1	21.7	16.3	11,560	17.9	21.7	13.1	19.8	16.3
Romania	5,158	23.7	25.8	19.5	22.4	25.0	6,425	20.3	18.6	17.1	22.5	23.0
Slovakia	4,101	27.5	32.7	28.0	30.3	19.5	4,300	27.6	28.0	29.9	27.3	24.8
Ukraine	4,539	29.9	38.2	31.6	26.0	17.5	5,422	15.7	18.5	18.6	15.8	7.5
South	34,425	21.5	23.6	22.9	22.6	17.2	35,873	22.4	25.8	23.6	21.9	18.8
Croatia	5,302	30.1	29.5	30.9	34.3	25.1	5,289	29.8	30.0	30.2	31.2	27.3
Greece	6,430	16.9	19.7	17.7	16.3	15.6	6,972	14.9	23.8	15.0	13.5	11.9
Italy	11,086	26.1	24.4	27.1	26.1	25.9	10,880	29.4	28.9	31.0	27.8	28.3
Malta	5,865	13.7	17.5	16.5	14.0	6.7	6,732	14.3	17.0	16.8	12.2	10.4
Slovenia	5,742	20.7	26.8	22.3	22.1	12.8	6,000	23.4	29.6	25.0	24.6	16.2
West	10,111	19.9	23.2	19.6	22.2	15.3	10,673	20.2	24.3	20.2	21.5	15.1
Belgium	2,838	17.5	24.3	16.6	19.1	11.7	2,715	15.2	21.8	15.4	15.2	10.1
France	4,269	22.8	24.3	22.6	26.2	18.7	4,712	25.5	27.7	23.0	30.3	21.3
Netherlands	3,004	19.5	21.0	19.5	21.3	15.5	3,246	19.9	23.3	22.3	19.0	13.9

^a^ Nonweighted and complete data were used for this table. Prevalence was calculated for each country-year combination, and then summary prevalences were calculated by taking averages of country- and year-specific prevalences to obtain the summary over years and countries. As such, the summary prevalences were not dependent on sample sizes and/or population sizes.

#### Study population.

The combined ESPAD data yielded a total of 286,075 participants aged 15–16 years. We excluded 35,749 individuals due to missing information on smoking status (*n* = 844) and/or SES (*n* = 35,067), resulting in a total study population of *n* = 250,326. Samples were nationally representative (except for Belgium, where the sample represents only Flanders), and the surveys were completed in classrooms under the supervision of a teacher or research assistant. Ethical approval was obtained separately for each country, following national ethical rules. Passive parental consent was used, unless active consent was imposed by ethical code. Detailed information on the sampling design, data collection, and ethical procedures is available in the ESPAD reports (24–27).

#### Variables.

Smoking was defined as “at least weekly smoking,” using the question, “How frequently have you smoked cigarettes during the last 30 days?” Participants who answered in the range from “less than one cigarette a day” to “more than 20 cigarettes a day” were classified as weekly smokers, while participants who answered “none” or “less than one cigarette a week” were considered nonsmokers (in that they did not smoke at least weekly).

SES was defined as the educational level of the most highly educated parent. Using 2 questions, participants were asked to report the “highest level of schooling completed” for their mother and father. Response options included “completed primary school or less,” “some secondary school,” “completed secondary school,” “some college or university,” “completed college or university,” “don’t know” or “does not apply.” “Don’t know” and “does not apply” responses were treated as missing. If the response for one parent was missing, only the information on the other parent was used. To account for differences in the distribution of parental education between countries, a country-specific parental education measure was constructed. Participants were assigned a rank based on the answer category of the most highly educated parent. As multiple individuals fell into the same category, all were assigned the mean rank of their category group. This rank was then divided by the number of respondents per country. This resulted in a continuous variable that could range from 0–1. In each country, 0 represents the lowest possible and 1 the highest possible parental educational level, while values between 0 and 1 represent the ordinal educational levels (with a 0.5 median). The same parental education variable was used in previous ESPAD publications (28, 29), and it was developed for calculating the slope index of inequality (SII) and the relative index of inequality (RII) (30).

We categorized countries into regions according to the United Nations geoscheme subregions of Europe (see Table 1). Regions are not fully represented by the available sample of countries; for example, only 3 countries in our data set are situated in Western Europe (a third of the countries from this region).

We created a “cluster” variable that corresponds to the smallest unit for which information was available for each participant: class, school, or country. This variable was used to account for the potential correlation of smoking outcomes of individuals within the same cluster, while not having to delete any additional observations due to missing data. Even if information about class, school, and country was available for every participant, not including all 3 levels as random effects might result in a more parsimonious model (31).

### Steps of the proposed method

In this section we explain each step of the methodology; Web Appendix 1 (available at https://doi.org/10.1093/aje/kwad029) provides additional details on the statistical methods used for each step. Steps 1, 2, and 3 create the graphical representation of linear trends in health inequalities across SES groups (objective 1). Steps 4 and 5 determine the likelihood of an eradication scenario (<5% prevalence in both high- and low-SES groups) if linear trends in inequalities continue (objective 2). The steps were applied to the data described above, as a proof of concept (objective 3). The code used for the analyses can be found in Web Appendixes 2–4.

#### 1. Estimation of smoking prevalence at both ends of the SES scale.

Multilevel logistic regression models were run using the *melogit* command in Stata, version 15.0 (StataCorp LLC, College Station, Texas). The analyses were performed separately for each survey year and gender, on the full sample and also stratified by European region. For all analyses, the variable “cluster” was used as the random effect, weekly smoking status was the dependent variable, and SES was the independent variable. Based on the estimated coefficients from these models, we estimated the weekly smoking prevalence for those at the lowest end of the SES scale (with SES = 0, henceforth referred to as P(0)) and for those at the highest end of the SES scale (with SES = 1, referred to as P(1)). Note that all individuals were included in the analysis, not just those at the upper and lower ends of the SES scale. We used a simulation-based approach to construct 95% confidence intervals (CIs) for the parameters estimated by P(0) and P(1). Although these 95% CIs could be constructed by using alternative methods (e.g., the Delta method, bootstrap), the calculation of the likelihood of eradication from step 5 requires the use of simulations, and these simulations easily provide these 95% CIs as a by-product. We simulated 10,000 values for the estimated coefficients from the models and then used them to calculate 10,000 simulated values for P(0) and P(1), separately for each survey year and gender.

#### 2. Prevalence points with 95% confidence regions.

We plotted the values of P(0) and P(1) against each other for each survey year. This resulted in a graph with 4 points: one point with coordinates (P(1), P(0)) for each survey year. Separate graphs were created for boys and girls, for all countries combined, and by European region. In the graphs, we wanted to describe the uncertainty around the prevalence points (P(1), P(0)). The 10,000 simulated values for the estimated coefficients from step 1 were used to create corresponding ellipse-shaped 95% confidence regions (CRs), separately for each survey year and gender. These 95% CRs were subsequently transformed to correspond to the prevalence points (P(1), P(0)).

#### 3. Fitting the regression line for the linear relationship between low- and high-SES prevalence.

The 4 points with coordinates (P(1), P(0)) were subsequently used to fit a linear regression line, which was presented in each graph. This regression line shows the linear relationship between low- and high-SES smoking prevalence over time. From this regression line we calculated the intercept (i.e., the point where the regression line crosses the *y*-axis), which indicates the estimated prevalence among low-SES adolescents when the prevalence among high-SES adolescents reached 0%.

#### 4. 95% confidence interval for the intercept of the regression line.

We used the simulated 10,000 values for the coordinates (P(1), P(0)) to fit 10,000 regression lines for the linear relationship between low- and high-SES smoking prevalence. Each one of these regression lines had an intercept, and we used these 10,000 intercept values to construct a 95% CI for the intercept of the linear relationship between low- and high-SES smoking prevalence. This 95% CI indicates the uncertainty around the value that the smoking prevalence among low-SES adolescents will have when the prevalence among high-SES adolescents is 0%.

#### 5. Likelihood of eradication.

In step 5 we used the 10,000 regression lines for the linear relationship between low- and high-SES smoking prevalence that were fitted in step 4. For each fitted regression line, we determined the intersection point with the axes; this may be the intercept if the *y*-axis is crossed first, or the point where the *x*-axis is crossed if the *x*-axis is crossed first. We calculated the likelihood of eradication as the percentage of the 10,000 fitted lines for which the intersection point fell within the desired scenario of <5% smoking prevalence among low- and high-SES adolescents (i.e., within 0.05 from the origin).

### Additional analysis of imputed data using sampling weights

For completeness, we also analyzed the data using weights after imputing the missing values. This analysis is presented separately, as this approach is not a requirement for the described methodology but a way to address the complex survey sampling design and the high number of missing values. We briefly describe how the imputation was performed and how the weights were constructed.

#### Imputation.

In order to perform the imputation of missing values, machine learning techniques were employed. Imputation was needed for 844 records with missing information on smoking status and 35,067 records with missing information on SES. Following Lantz (32), different prediction algorithms were tried and diagnosed. In order to choose the best one, a *k*-fold cross-validation (33) was performed, dividing the complete data into 100 parts, which were recursively predicted using the observations excluded from the selected. Subsequently, diagnostics of each prediction method were averaged in order to obtain an approximation of the model’s goodness of fit. A regression tree model with recursive partitioning (34) was chosen, which classifies the observations splitting the data set into subsamples according to several individual and environmental characteristics, such as family status, friendship relationships, and substance consumption habits of each individual. The tree was pruned in order to reduce its complexity and the risk of overfitting (35), and to ensure high accuracy of the prediction process (36). Indeed, predictions for smoking status exhibit an accuracy (i.e., proportion of observations correctly predicted) ranging from 89.59% and 96.21%, with a mean of 92.70% and a standard deviation of 1.5%. In the case of SES, which is a continuous variable, it is not possible to exactly compute the value of accuracy, but the lowest value of root mean squared error obtained for actual and predicted values was 0.0845.

#### Weights.

A poststratification weighting approach was applied to adjust some national samples/years to the sociodemographic composition of the respective target populations. This correction technique assigns a differential weight to each survey respondent: Those from underrepresented groups are weighted more than 1, and those from overrepresented groups get a weight smaller than 1. Therefore, where necessary due to the nonproportional allocation of the sample to stratification variables and possible differences in response rates, sampling weights were calculated by the ESPAD principal investigators (e.g., to account for gender, geographical distribution, and type and size of school) and added to the ESPAD databases. Further details about geographical coverage, sampling procedure in each country, representativeness, and characteristics of the samples, as well as participation rates and sampling weights, can be found in the relevant ESPAD Reports (24–27).

## RESULTS

Table 1 presents a description of the number of participants included in the study and the smoking prevalence across years and countries. The smoking prevalence across countries and survey years was 22.2% among boys and 21.4% among girls. In 2003 the prevalence was 26.6% among boys and 25.0% among girls, and in 2015 it was 16.1% and 16.2%, respectively. While the 2015 smoking prevalence in Eastern Europe was 20.8% among boys and 21.2% among girls, Northern Europe showed rates of 11.6% and 10.5%, respectively.

**Figure 1:**
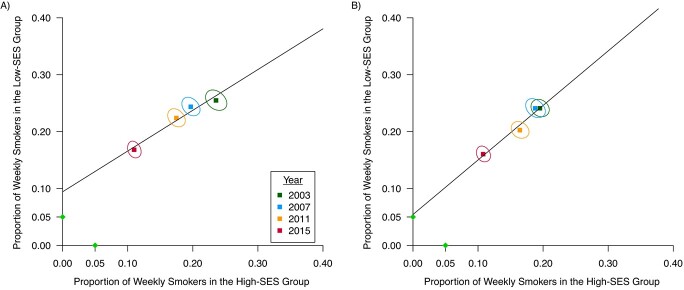
Linear trend in socioeconomic inequalities in smoking in 23 European countries among boys (A) and girls (B), with fitted regression line and 95% confidence regions for each time point. Data from 23 European countries of the European Survey Project on Alcohol and Other Drugs in 4 survey waves (2003–2015). The proportion of weekly smokers in the low–socioeconomic status (SES) group is P(0) and in the high-SES group is P(1). The green dots indicate the point of reaching <5% prevalence, which is defined as the eradication scenario.

Figure 1 presents the prevalence points, their corresponding 95% CRs, and the fitted regression line. Among both boys and girls, the declining line from 2003 to 2015 reflects a downward trend in smoking for the high- and low-SES groups. The decrease is larger between 2015 and 2011 than between the previous survey years. The downward trend is similar for boys (Figure 1A) and girls (Figure 1B). Smoking prevalence decreased more slowly among low-SES than among high-SES adolescents, as the slope indicated that prevalence would decrease with 0.72% among low-SES boys and 0.92% among low-SES girls, for each 1% decrease among high-SES youth. Furthermore, the regression lines show that, with continuing trends, the smoking prevalence would be approximately 9% among low-SES boys and 5% among low-SES girls when the prevalence reached 0% for their high-SES counterparts.

Table 2 describes the estimated intercepts as presented in Figure 1. The smoking prevalence among low-SES boys was estimated to be 9.4% (95% CI: 6.1, 12.7) when 0.0% for high-SES boys. Prevalence among low-SES girls was estimated to be 5.4% (95% CI: 1.4, 9.2) with a prevalence of 0.0% among high-SES girls. The likelihood of eradication was <1% among boys and 37% among girls, which is consistent with the differences in estimated intercepts. Web Table 1 shows in more detail the estimated parameter values and estimated variance-covariance matrix from multilevel logistic regression models, stratified by region, gender, and survey year. Web Table 2 additionally presents the estimated smoking prevalence among low- and high-SES adolescents based on multilevel logistic regression models.

**Table 2:** The Estimated Intercept of the Regression Line, Simulation-Based 95% Confidence Interval for the Intercept, and Simulation-Based Value for the Likelihood of Eradication of Smoking, Stratified by Gender, for All Countries, and Stratified by Region, Using Data from 23 European Countries, European Survey Project on Alcohol and Other Drugs, Over 4 Survey Waves, 2003–2015

**Regions**	**Boys**			**Girls**		
	**Intercept** ^**a**^	**95% CI**	**Eradication** ^**b**^ **, %**	**Intercept** ^**a**^	**95% CI**	**Eradication** ^**b**^ **, %**
All countries	9.4	6.1, 12.7	<1	5.4	1.4, 9.2	37
North	7.2	2.8, 11.4	14	8.5	5.3, 12.1	2
East	15.3	10.1, 20.5	0	11.1	4.3, 17.7	3
South	10.5	3.2, 17.5	6	3.4	7.0, 11.8	54
West	8.8	−5.2, 21.3	18	−8.8	−38.7, 21.3	48

Abbreviations: CI, confidence interval; SES, socioeconomic status.

^a^ Intercept of the regression line (i.e., value of smoking prevalence among low-SES adolescents if smoking prevalence among those with high SES is 0%), as presented in Figure 1 and Web Figures 1–4.

^b^ Eradication defined as prevalence <5% across the socioeconomic spectrum. Likelihood of eradication was calculated as the percentage of simulated regression lines with values that cross the *x*-axis or *y*-axis within the range of (0,0.05) and (0.05,0), as indicated in the graphs presented in Figure 1 and Web Figures 1–4.

Table 2 also stratifies the results by region, with graphical representations available in Web Figures 1–4. Trends seem more favorable in countries from Northern and Western Europe than in countries from Southern and Eastern Europe. The estimated intercepts for the Northern and Western European countries range between 7.2 and 8.8, while the estimated intercepts tended to be higher for Eastern and Southern European countries. Correspondingly, the likelihood of eradication is generally higher for Northern and Western European countries. For countries from Western Europe, the trend tended to favor low-SES girls over high-SES girls. However, the larger 95% CRs indicate much more uncertainty around these estimates.

The analyses using imputed and weighted data are presented in Web Tables 3 and 4 and Web Figure 5. Web Table 3 presents the weighted smoking prevalences by gender, year, and country. Prevalences were generally somewhat higher in girls. Web Table 4 presents the estimated intercepts and likelihoods of eradication using the imputed data analyzed using the weights. With the exception of the Eastern European countries, the intercepts were generally lower and likelihoods of eradication higher compared with the main analysis. The same patterns as seen in the main results were observed: Likelihood of eradication was higher for girls than boys and for Northern and Western European countries compared with Southern and Eastern European countries.

## DISCUSSION

### Key findings

We present a novel method of describing and interpreting linear trends in health inequalities. The method models the linear relationship between prevalences in high-SES and low-SES groups and evaluates the likelihood of eradication of SES inequalities by using a simulation-based approach. We provided a proof of concept by applying this method to the empirical data on smoking. We found that smoking prevalence in Europe decreased more slowly among low-SES than among high-SES adolescents. Prevalence in boys was estimated to be 9.4% for low SES when 0.0% for high SES, and 5.4% in girls when 0.0% for high SES. The likelihood of simultaneous eradication in low- and high-SES youth was <1% among boys and 37% among girls. Trends seem more favorable in Northern and Western Europe than in Southern and Eastern Europe.

### Strengths and limitations of the data

The ESPAD data set provides a large sample of adolescents from Europe, over multiple waves. The sampling and surveying methods are highly standardized across countries and over time, which makes the data very suitable for trend studies and regional comparisons. ESPAD data also allow measurement of adolescents’ socioeconomic background, by including parental educational level. Even though some adolescents may misclassify their parents’ education, adolescent-reported parental education has been found to be strongly associated with adolescent smoking (37) and therefore serves as a relevant socioeconomic predictor of smoking in this population.

The data may not be generalizable to the entire adolescent population of Europe, as countries from the West and South are underrepresented in our data set. Although samples are largely representative, results may not fully generalize to the adolescent population of the included countries because a limited number of schools were included, only school-attending adolescents were included, and response rates ranged between 78% and 98% (mean = 87%) (24–27). In an additional analysis we used weights and imputed the missing data. The results showed a higher likelihood of eradication for most European regions; however, no substantive inferences should be made based on these modified data. However, potentially limited generalizability would not affect the internal validity of changes over time in smoking prevalence, as the survey method was identical between survey waves.

### Strengths and limitations of the methodology

This proof-of-concept analysis demonstrated that, by modeling the relationship between prevalence in high- and low-SES groups, we can quantify the likelihood of eradication of socioeconomic inequalities. This provides a novel approach for monitoring inequalities from which insights can be derived beyond the magnitude of inequality.

There are, however, some limitations to this methodology that may require further development of the methods, after the present proof of concept.

First, this methodology assumes a linear trend over time in smoking prevalence for high- and low-SES groups. Although this approach will capture the linear component of the true relationship, this assumption may not hold in general. In many countries we observed a larger decrease in smoking between 2011 and 2015 than between earlier survey waves, especially among low-SES groups. If future trends follow the trends since 2015, the eradication scenario of <5% smokers across SES groups may be more likely than predicted by this study. Further development of the methodology may need to consider how to incorporate quadratic or other nonlinear terms into the regression models, without compromising the interpretability of the results. These models will require data from a larger number of survey waves.

Second, the present method focusses on the relationship between SES and the log odds of smoking across all SES groups, and does not consider specific groups individually. The ranking methods gives less weight to groups with intermediate SES. We cannot rule out the possibility that these groups have more favorable or unfavorable smoking trends than the groups at the extremes. This limits the method’s use in monitoring eradication for all SES categories (4).

Third, the analysis requires a relatively high number of respondents or countries to yield sufficiently reliable results. Analyses of single countries were not presented because the confidence regions became extremely large. The method may need to be further optimized for use with smaller data sets.

### Applicability of the methodology

We developed this methodology with the aim of providing a tool for epidemiologists to describe health inequalities. We demonstrated the applicability to smoking and provided new insights into the likelihood of eradication of smoking. However, there are important issues to consider when further applying this method.

First, the graphs do not necessarily present the trend in the order of the survey waves over time. If smoking prevalence would have increased in both low- and high-SES groups, either temporally or consistently, the regression line would look similar in the visual presentation. Trend scenarios can be derived only if the trend is consistently declining over time and at both ends of the SES scale.

Second, this methodology may be applied to inequalities on topics other than smoking. The method may, however, be less suitable for beneficial health outcomes, as these should increase for the desired health effect. However, it may be possible to determine the desired level for such behavior—for example, minimum levels of physical activity or vegetable consumption—and use these as the 0 value on a reversed scale. Moreover, many health outcomes generally require reduction, such as alcohol use, body mass index, sedentary behavior, and various disease outcomes. For some of these, the desired value would need to be changed from 5% to a suitable value.

Third, the analysis is relatively complex and therefore will not be equally accessible to every epidemiologist or public health researcher. A simplified alternative would be to plot prevalence rates for the highest and lowest SES groups, and to judge the trend qualitatively based on this visualization. Such a simplified method may already improve the interpretation of the trends when compared with traditional methods based on inequalities indices.

### Interpretation of the empirical results

Our results show that it is unlikely that Europe will reach a <5% smoking prevalence at the same time among low-SES and high-SES adolescents, especially among boys, if trends continue to follow the current pattern. Many policies that have been implemented in the past decades, such as smoke-free policies, advertising bans, and school-based prevention, were more effective among high-SES youth than low-SES youth or were equally effective in both groups at best (38). Policies and interventions are needed that are especially effective in youth with lower socioeconomic backgrounds, which may include tobacco tax increases (38) and school smoke-free policies (39).

Trends in inequalities in smoking were more favorable in Northern and Western Europe than in Southern and Eastern Europe, although results in Western Europe seemed less robust due to the low number of countries. These geographical patterns are in line with expectations from the tobacco epidemic, as described by Lopez et al. (40). Smoking prevalence in Northern and Western Europe started to decrease earlier, and inequalities in smoking emerged and consolidated earlier, than in Eastern and Southern Europe.

## CONCLUSIONS

This paper proposes a novel methodology and provides a proof-of-concept analysis for the visualization of linear trends in health inequalities and quantification of a scenario of eradication. We have outlined challenges that may need to be addressed in further development and application of this method. For adolescent smoking in Europe, we conclude that the current trend is unlikely to lead to smoking eradication at the same time among low-SES and high-SES adolescents.

## Supplementary Material

Web_Material_kwad029
