# Supplementary material for: A Novel Methodological Approach to Measure Linear Trends in Health Inequalities: Proof of Concept With Adolescent Smoking in Europe
Source: Am J Epidemiol. 2023 Feb 3;192(6):963–71. doi: 10.1093/aje/kwad029 (PMC10516356; doi:10.1093/aje/kwad029)
Supplement: Web_Material_kwad029 [file web_material_kwad029.pdf]

WEB MATERIAL

A Novel Methodological Approach to Measure Linear Trends in Health Inequalities: Proof-of-Concept Analysis of Adolescent Smoking In Europe

Mirte A.G. Kuipers, Kaidi Kang, Anca D. Dragomir, Karin Monshouwer, Elisa Benedetti, Gabriele Lombardi, George Luta, Anton E. and Kunst

Table of contents

Web Table 1: Estimated parameter values and estimated variance-covariance matrix from multilevel logistic regression models, stratified by region, gender and survey year. See Box S1 for interpretations of the parameters. 2

Web Table 2: Estimated smoking prevalence (in %, with simulation-based 95% CIs) among low and high SES adolescents based on multilevel logistic regression models, by gender and survey year, for all countries and by region. 3

Web Table 3: Weighed smoking prevalence on imputed data in 23 European countries over four survey waves. For the totals (across countries and years), average smoking prevalence across countries and/or years is presented, to which all countries/years contribute equally[a]. (Weighted prevalences derived from imputed data)........ 4

Web Table 4: The estimated intercept of the regression line, simulation-based 95% confidence interval for the intercept, and simulation-based value for the likelihood of eradication of smoking, stratified by gender, for all countries and stratified by region. (Results derived from weighted and imputed data)........ 5

Web Figure 1: NORTHERN EUROPE: Linear trend in socioeconomic inequalities in smoking among boys (**A.**) and girls (**B.**), with fitted regression line and 95% confidence regions for each time point.... 6

Web Figure 2: EASTERN EUROPE: Linear trend in socioeconomic inequalities in smoking among boys (**A.**) and girls (**B.**), with fitted regression line and 95% confidence regions for each time point. 7

Web Figure 3: SOUTHERN EUROPE: Linear trend in socioeconomic inequalities in smoking among boys (**A.**) and girls (**B.**), with fitted regression line and 95% confidence regions for each time point.... 8

Web Figure 4: WESTERN EUROPE: Linear trend in socioeconomic inequalities in smoking among boys (**A.**) and girls (**B.**), with fitted regression line and 95% confidence regions for each time point. 9

Web Figure 5: Linear trend in socioeconomic inequalities in smoking in 23 European countries among boys (**A.**) and girls (**B.**), with fitted regression line and 95% confidence regions for each time point. (Results derived from weighted and imputed data) 10

Web Appendix 1: Details regarding the statistical methods used for each step of the proposed methodology. 11

Web Appendix 2: Unweighted analyses in Stata 13

Web Appendix 3: Weighted analysis in Stata 16

Web Appendix 4: R code........ 19

Web Table 1: Estimated parameter values and estimated variance-covariance matrix from multilevel logistic regression models, stratified by region, gender and survey year. See Web Appendix 1for interpretations of the parameters. Data from 23 European countries of the ESPAD study in four survey waves (2003-2015).

	**Boys**				**Girls**			
	**2003**	**2007**	**2011**	**2015**	**2003**	**2007**	**2011**	**2015**
**All countries**								
$\hat{b}_0$	-1.073	-1.133	-1.243	-1.599	-1.147	-1.148	-1.370	-1.656
$\hat{b}_1$	-0.103	-0.273	-0.310	-0.493	-0.270	-0.316	-0.257	-0.457
$\widehat{\text{Var}}(\hat{b}_0)$	0.001	0.001	0.001	0.002	0.001	0.001	0.001	0.002
$\widehat{\text{Var}}(\hat{b}_1)$	0.004	0.003	0.004	0.005	0.003	0.004	0.004	0.005
$\widehat{\text{Cov}}(\hat{b}_0, \hat{b}_1)$	-0.002	-0.002	-0.002	-0.002	-0.001	-0.002	-0.002	-0.002
**North**								
$\hat{b}_0$	-1.104	-1.150	-1.283	-2.071	-1.134	-1.194	-1.563	-1.892
$\hat{b}_1$	-0.321	-0.496	-0.799	-0.627	-0.598	-0.703	-0.524	-1.311
$\widehat{\text{Var}}(\hat{b}_0)$	0.005	0.005	0.005	0.009	0.004	0.005	0.005	0.009
$\widehat{\text{Var}}(\hat{b}_1)$	0.012	0.012	0.014	0.024	0.010	0.015	0.014	0.025
$\widehat{\text{Cov}}(\hat{b}_0, \hat{b}_1)$	-0.006	-0.006	-0.006	-0.011	-0.004	-0.007	-0.006	-0.011
**East**								
$\hat{b}_0$	-0.855	-0.965	-0.960	-1.292	-1.279	-1.152	-1.146	-1.447
$\hat{b}_1$	-0.035	-0.302	-0.310	-0.603	-0.063	-0.055	-0.152	-0.374
$\widehat{\text{Var}}(\hat{b}_0)$	0.003	0.005	0.004	0.004	0.004	0.004	0.004	0.004
$\widehat{\text{Var}}(\hat{b}_1)$	0.008	0.012	0.010	0.010	0.007	0.011	0.011	0.009
$\widehat{\text{Cov}}(\hat{b}_0, \hat{b}_1)$	-0.004	-0.006	-0.005	-0.005	-0.004	-0.005	-0.005	-0.004
**South**								
$\hat{b}_0$	-1.253	-1.160	-1.420	-1.532	-1.037	-1.048	-1.421	-1.560
$\hat{b}_1$	0.056	-0.086	-0.054	-0.372	-0.025	-0.170	-0.163	-0.116
$\widehat{\text{Var}}(\hat{b}_0)$	0.005	0.004	0.005	0.007	0.005	0.004	0.005	0.008
$\widehat{\text{Var}}(\hat{b}_1)$	0.013	0.010	0.013	0.018	0.012	0.009	0.012	0.020
$\widehat{\text{Cov}}(\hat{b}_0, \hat{b}_1)$	-0.007	-0.005	-0.006	-0.009	-0.006	-0.004	-0.006	-0.010
**West**								
$\hat{b}_0$	-1.182	-1.372	-1.421	-1.808	-0.836	-1.298	-1.282	-1.820
$\hat{b}_1$	-0.308	-0.382	0.098	-0.208	-0.687	-0.470	-0.241	-0.166
$\widehat{\text{Var}}(\hat{b}_0)$	0.017	0.015	0.016	0.022	0.014	0.015	0.014	0.023
$\widehat{\text{Var}}(\hat{b}_1)$	0.048	0.040	0.044	0.052	0.043	0.044	0.038	0.057
$\widehat{\text{Cov}}(\hat{b}_0, \hat{b}_1)$	-0.023	-0.019	-0.022	-0.027	-0.019	-0.020	-0.017	-0.029

Web Table 2: Estimated smoking prevalence (in %, with simulation-based 95% CIs) among low and high SES adolescents based on multilevel logistic regression models, by gender and survey year, for all countries and by region. Data from 23 European countries of the ESPAD study in four survey waves (2003-2015).

	Boys				Girls			
	**2003**	**2007**	**2011**	**2015**	**2003**	**2007**	**2011**	**2015**
**All countries**								
High SES	23.6 (22.9-25.2)	19.7 (18.3-21.1)	17.5 (16.2-18.8)	11.0 (10.0-12.1)	19.5 (18.1-20.9)	18.8 (17.3-20.3)	16.4 (15.1-17.8)	10.8 (9.8-11.9)
Low SES	25.5 (23.7-27.3)	24.4 (22.8-26.0)	22.4 (20.8-24.0)	16.8 (15.4-18.3)	24.1 (22.6-25.7)	24.1 (22.5-25.8)	20.3 (18.8-21.8)	16.0 (14.7-17.5)
**North**								
High SES	19.4 (16.9-22.2)	16.2 (14.0-18.6)	11.1 (9.3-13.1)	6.3 (5.0-8.0)	15.0 (13.1-17.2)	13.0 (11.0-15.4)	11.0 (9.2-13.1)	3.9 (3.0-5.1)
Low SES	24.9 (21.9-28.1)	24.0 (21.1-27.2)	21.7 (18.9-24.8)	11.2 (9.0-13.8)	24.3 (21.8-27.1)	23.2 (20.4-26.3)	17.3 (15.0-20.0)	13.1 (10.7-16.0)
**East**								
High SES	29.1 (26.4-32.0)	22.0 (19.4-24.8)	21.9 (19.5-24.5)	13.1 (11.4-14.9)	20.7 (18.4-23.3)	23.0 (20.2-26.1)	21.5 (18.9-24.3)	13.9 (12.2-15.9)
Low SES	29.8 (27.0-32.9)	27.6 (24.5-31.0)	27.7 (24.8-30.8)	21.6 (19.1-24.2)	21.8 (19.4-24.4)	24.0 (21.2-27.1)	24.1 (21.4-27.0)	19.0 (16.9-21.4)
**South**								
High SES	23.2 (20.3-26.4)	22.3 (19.9-25.0)	18.6 (16.1-21.4)	13.0 (10.8-15.4)	25.7 (22.5-29.2)	22.8 (20.3-25.6)	17.0 (14.6-19.8)	15.8 (13.1-18.9)
Low SES	22.2 (19.3-25.4)	23.9 (21.2-26.7)	19.5 (16.8-22.4)	17.8 (14.9-21.0)	26.2 (23.1-29.5)	26.0 (23.3-28.9)	19.4 (16.9-22.2)	17.4 (14.5-20.7)
**West**								
High SES	18.4 (13.9-24.0)	14.8 (11.2-19.1)	21.0 (16.3-26.8)	11.8 (8.7-15.7)	17.9 (13.6-23.1)	14.6 (10.9-19.2)	17.9 (13.7-23.0)	12.1 (8.7-16.6)
Low SES	23.5 (18.1-29.7)	20.2 (15.9-25.4)	19.5 (15.0-24.8)	14.1 (10.3-19.0)	30.2 (24.5-36.6)	21.5 (16.9-26.9)	21.7 (17.2-27.0)	13.9 (10.0-19.0)

Web Table 3: Weighed smoking prevalence on imputed data in 23 European countries of the ESPAD study in four survey waves (2003-2015). For the totals, average smoking prevalence across countries and/or years is presented, to which all countries/years contribute equally[a]. (Weighted prevalences derived from imputed data)

	Boys						Girls					
	N	Smoking prevalence (%)					N	Smoking prevalence (%)				
	2003-2015	2003-2015	2003	2007	2011	2015	2003-2015	2003-2015	2003	2007	2011	2015
**Total all countries**	140212	22.9	27.4	23.2	23.3	16.4	137809	22.0	25.6	22.9	22.3	16.5
Denmark	3420	18.5	21.3	23.5	19.9	10.1	3812	19.0	23.5	26.1	15.3	13.7
Estonia	4841	24.1	33.5	25.6	21.2	15.9	4874	19.8	26.7	18.0	21.6	12.9
Finland	7590	22.1	27.0	22.6	24.1	15.9	8410	22.7	31.4	22.4	24.5	14.2
Iceland	6538	9.5	15.5	11.7	5.9	3.1	6281	10.5	15.3	13.8	7.2	4.5
Latvia	4365	34.5	37.1	36.1	37.1	18.8	4467	26.2	25.4	29.5	28.5	15.5
Lithuania	6223	33.3	42.5	30.3	30.6	20.7	6265	21.3	23.6	20.3	23.6	15.3
Norway	6515	10.2	17.1	10.5	7.1	3.9	6234	13.8	24.4	15.1	6.8	3.6
Sweden	5702	10.4	10.1	11.1	14.0	6.3	5809	14.6	17.3	15.3	14.9	9.9
**Total North**	45194	20.3	25.5	21.4	20.0	11.8	46152	18.5	23.5	20.0	17.8	11.2
Bulgaria	5024	29.5	35.9	30.8	26.4	25.3	5134	36.8	42.1	37.7	37.7	30.4
Czech Republic	6481	31.0	36.5	30.4	34.3	21.4	7220	35.2	37.3	38.8	35.6	27.1
Hungary	5936	27.1	31.4	25.8	29.0	21.8	5788	27.0	30.4	27.0	28.8	21.7
Poland	12327	21.4	27.3	16.6	22.4	17.0	13391	17.2	19.6	12.9	18.0	16.8
Romania	5794	23.8	25.8	20.3	22.2	25.1	7088	20.5	18.4	18.1	22.7	23.2
Slovakia	4329	27.5	32.6	28.1	29.7	20.4	4478	27.7	28.5	29.7	27.8	24.6
Ukraine	5135	32.2	41.7	32.7	27.1	19.4	5986	16.5	20.3	19.0	16.5	8.2
**Total East**	45026	27.5	33.0	26.4	27.3	21.5	49085	25.9	28.1	26.2	26.7	21.7
Croatia	5793	30.6	30.4	31.4	34.4	25.9	5627	30.1	30.6	30.4	31.7	27.1
Greece	6810	17.3	20.0	17.9	16.7	16.5	7251	15.6	23.6	14.9	15.3	11.6
Italy	12145	26.7	24.8	27.8	27.0	25.8	11550	29.6	28.9	31.4	27.9	28.1
Malta	6585	13.9	17.3	16.9	14.4	7.1	7229	14.7	17.3	17.2	12.7	10.6
Slovenia	6206	21.0	27.2	22.6	22.2	13.2	6307	23.3	29.5	24.8	24.6	16.2
**Total South**	37539	21.9	23.9	23.3	22.9	17.7	37964	22.6	26.0	23.8	22.4	18.7
Belgium	3504	18.1	26.0	18.0	19.4	11.4	3245	16.5	23.0	16.5	17.2	10.8
France	5089	21.7	24.4	21.0	26.1	16.3	5390	25.3	28.3	21.1	31.9	20.9
Netherlands	3860	21.7	24.5	22.3	23.8	15.1	4027	21.6	24.2	25.8	21.4	13.4
**Total West**	12453	20.5	25.0	20.4	23.1	14.3	12662	21.1	25.2	21.1	23.5	15.0

[a] Prevalence was calculated for each country-year combination, and then summary prevalences were calculated by taking averages of country and year specific prevalences to obtain the summary over years and countries. As such, the summary prevalences were not dependent on sample sizes and/or population sizes.

Web Table 4: The estimated intercept of the regression line, simulation-based 95% confidence interval for the intercept, and simulation-based value for the likelihood of eradication of smoking, stratified by gender, for all countries and stratified by region. Data from 23 European countries of the ESPAD study in four survey waves (2003-2015). (Results derived from weighted and imputed data)

	Boys	Girls
**All countries**		
Intercept and 95% CI[a]	4.8 (1.3;8.2)	3.7 (-1.0;7.8)
Likelihood of eradication[b]	51%	68%
**North**		
Intercept and 95% CI[a]	4.1 (0; 7.6)	7.1 (4.3;10.4)
Likelihood of eradication[b]	66%	7%
**East**		
Intercept and 95% CI[a]	16.5 (10.7;22.1)	14.2 (5.3;22.1)
Likelihood of eradication[b]	0%	1%
**South**		
Intercept and 95% CI[a]	7.5 (0;14.9)	1.0 (-11.1;10.5)
Likelihood of eradication[b]	20%	65%
**West**		
Intercept with simulated 95% CI[a]	5.0 (-9.4;17.4)	-6.7 (-28.5; 11.6)
Likelihood of eradication[b]	34%	61%

[a] Intercept of the regression line (i.e. value of smoking prevalence among low SES if smoking prevalence among high SES is 0%), as presented in Figure 1 and Figures S1-S4.

[c] Eradication defined as prevalence <5% across the socioeconomic spectrum. Likelihood of eradication was calculated as the percentage of simulated regression lines with values that cross the x-axis or y-axis within the range of (0,0.05) and (0.05,0), as indicated in the graphs presented in Figure 1 and Figures S1-S4.

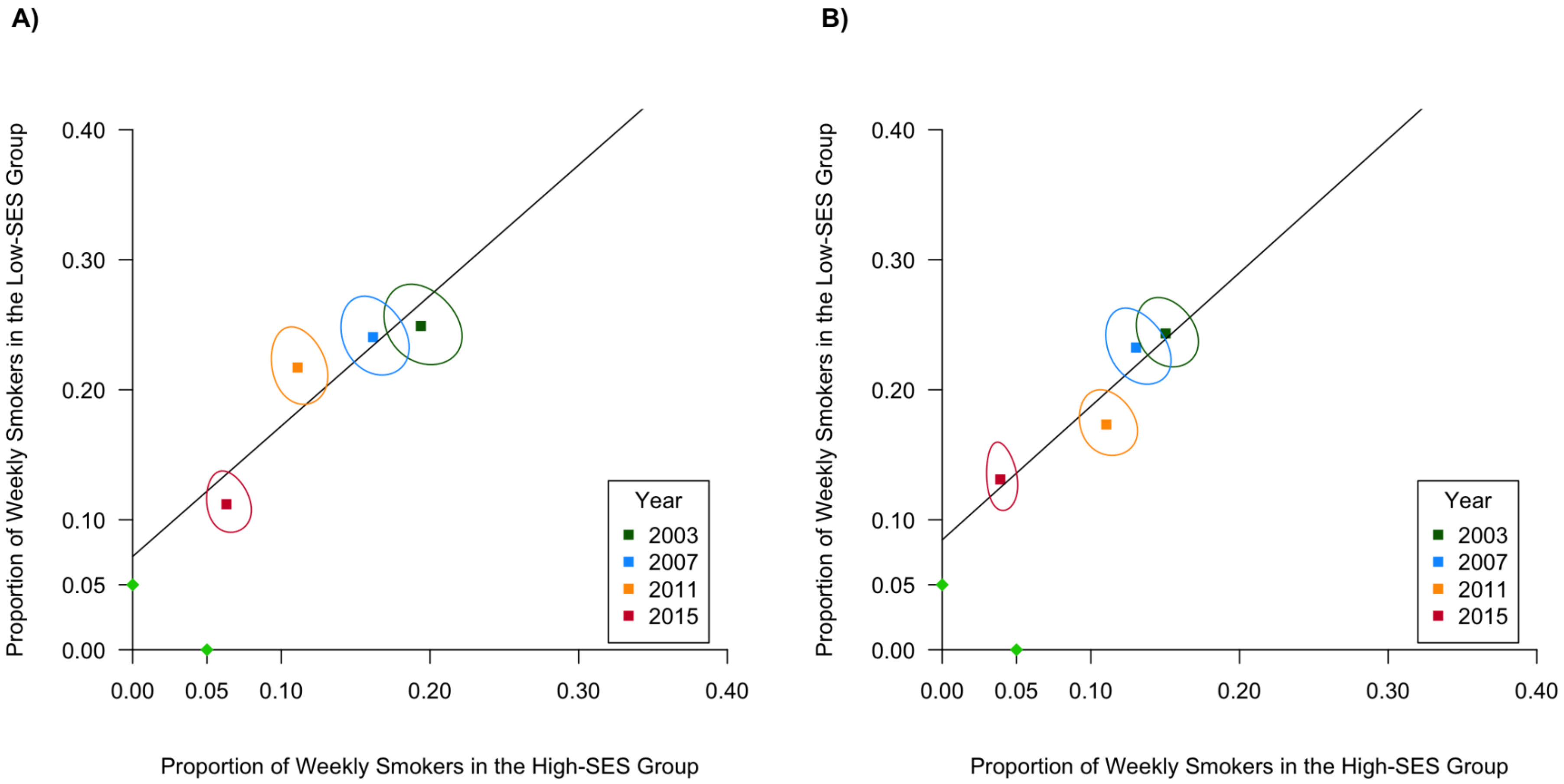

Web Figure 1: NORTHERN EUROPE: Linear trend in socioeconomic inequalities in smoking among boys (**A.**) and girls (**B.**), with fitted regression line and 95% confidence regions for each time point. Data from 8 European countries of the ESPAD study in four survey waves (2003- 2015).

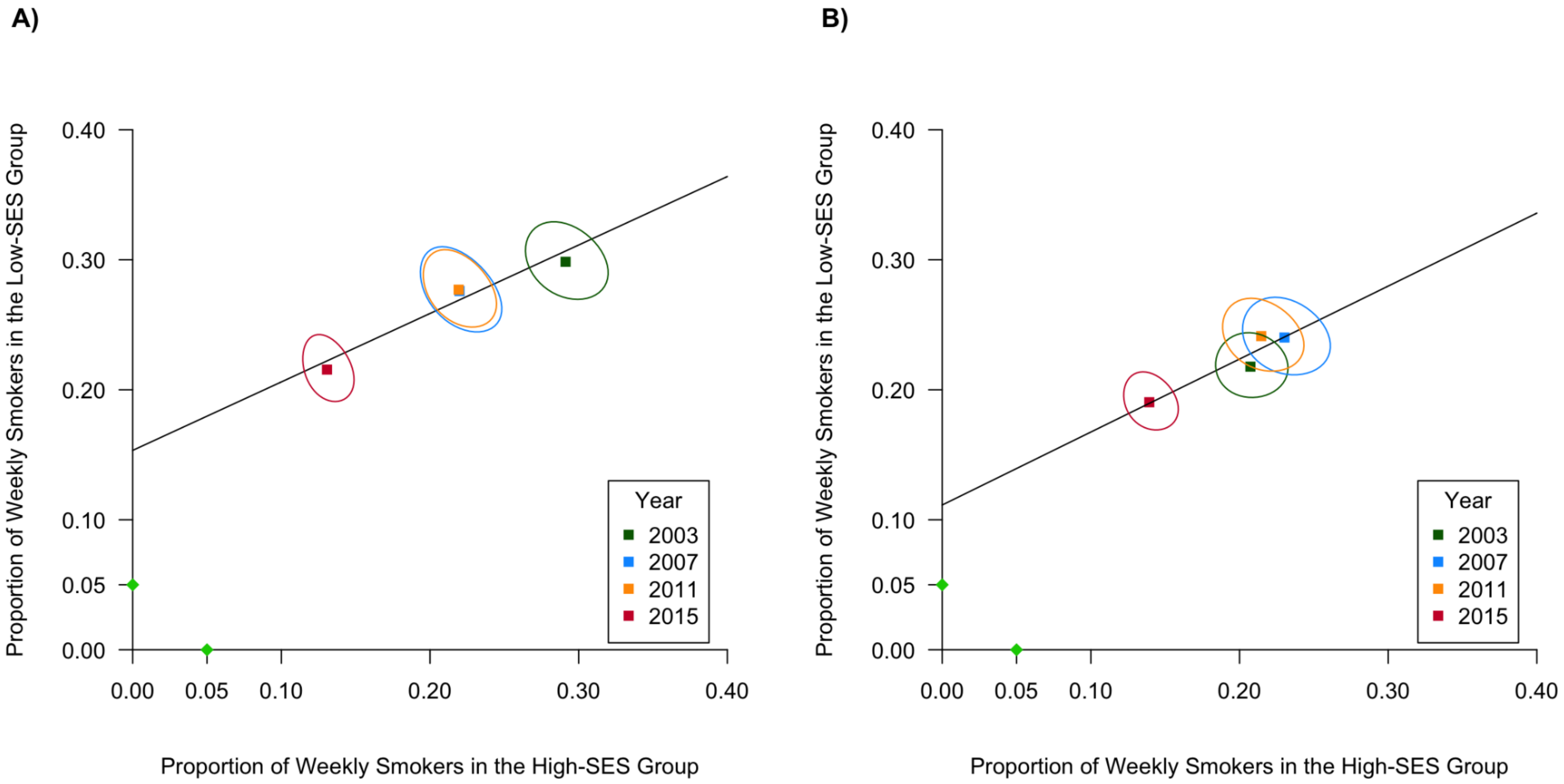

Web Figure 2: EASTERN EUROPE: Linear trend in socioeconomic inequalities in smoking among boys (**A.**) and girls (**B.**), with fitted regression line and 95% confidence regions for each time point. Data from 7 European countries of the ESPAD study in four survey waves (2003- 2015).

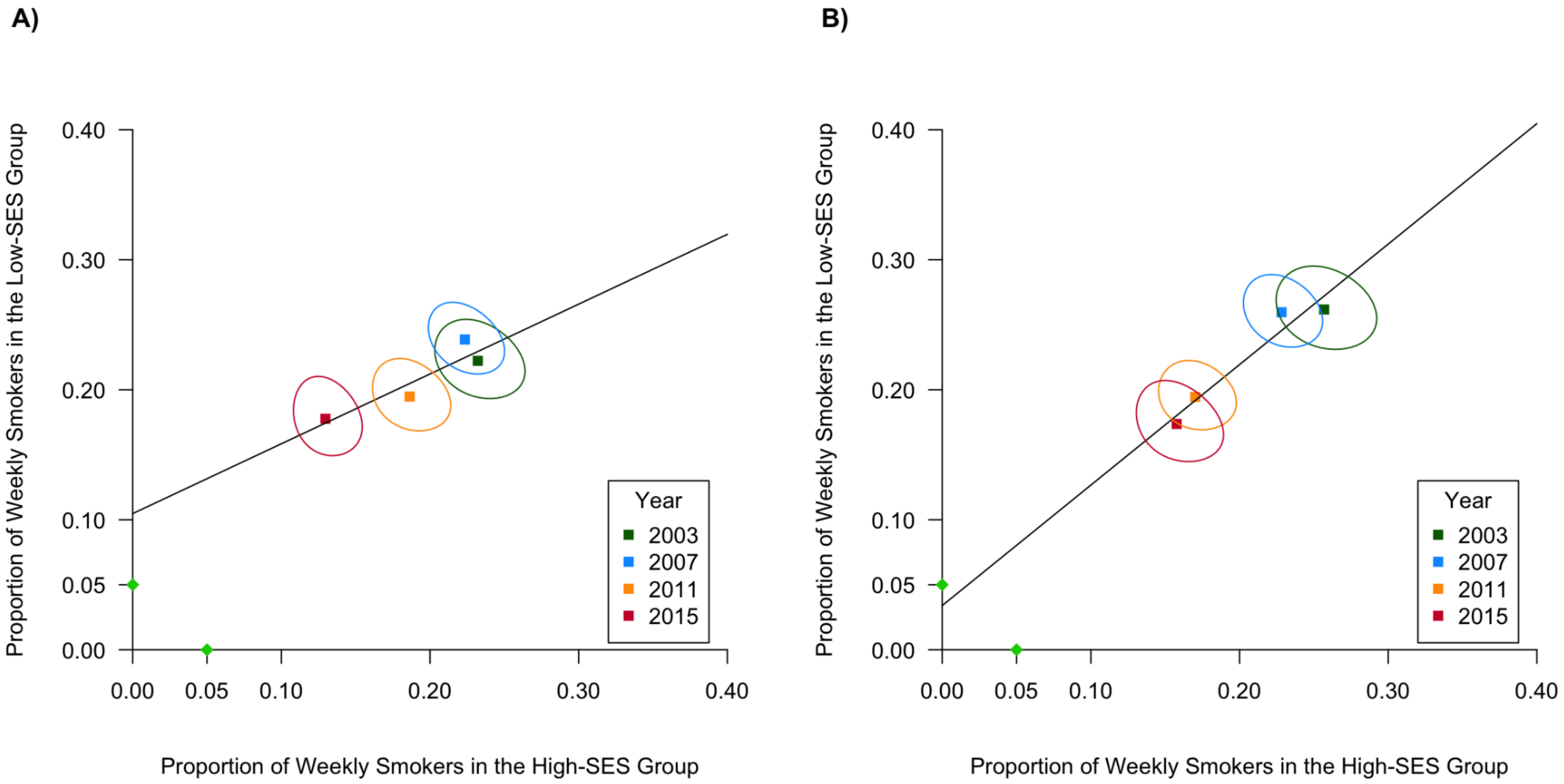

Web Figure 3: SOUTHERN EUROPE: Linear trend in socioeconomic inequalities in smoking among boys (**A.**) and girls (**B.**), with fitted regression line and 95% confidence regions for each time point. Data from 5 European countries of the ESPAD study in four survey waves (2003- 2015).

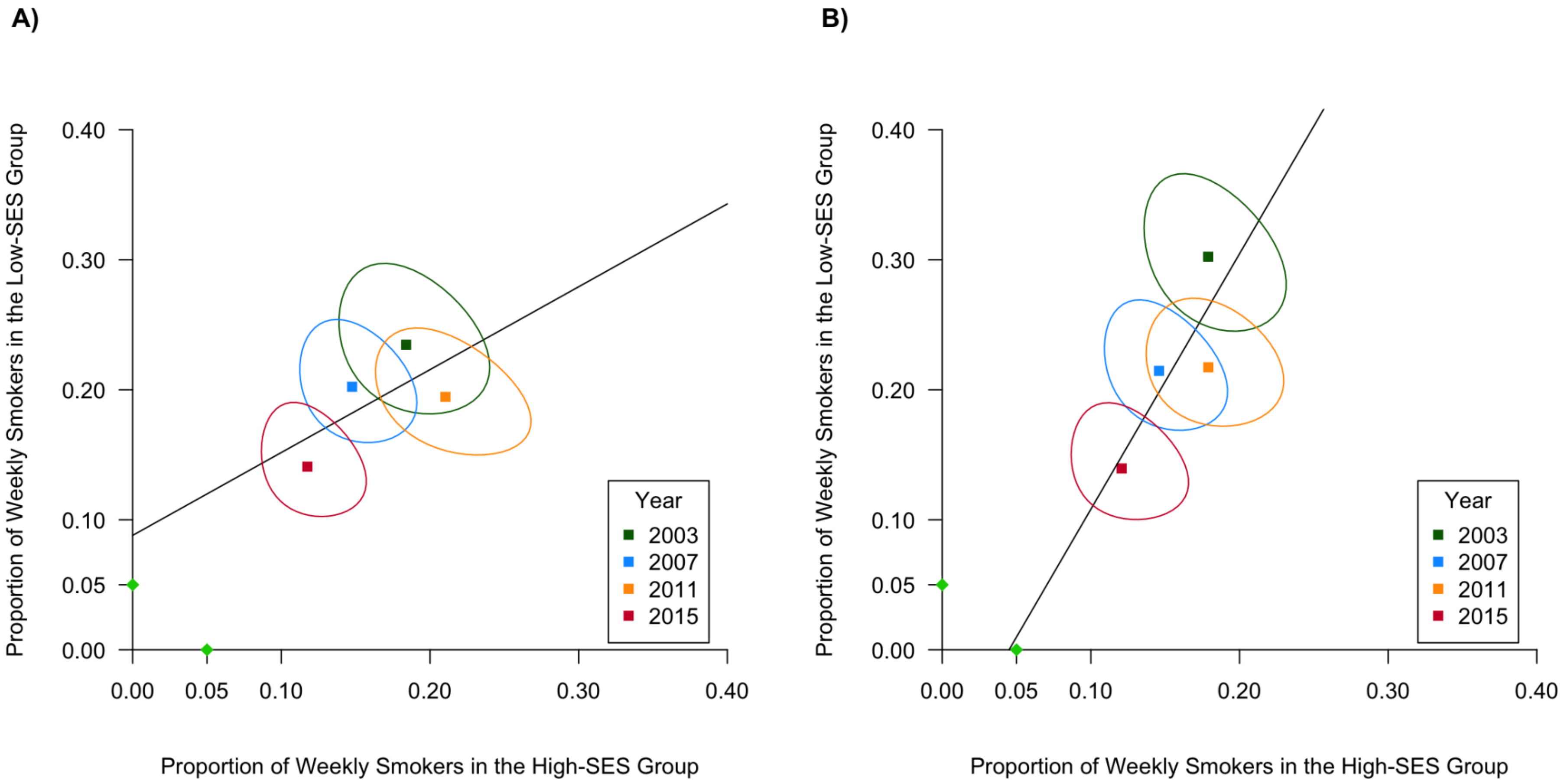

Web Figure 4: WESTERN EUROPE: Linear trend in socioeconomic inequalities in smoking among boys (**A.**) and girls (**B.**), with fitted regression line and 95% confidence regions for each time point. Data from 3 European countries of the ESPAD study in four survey waves (2003- 2015).

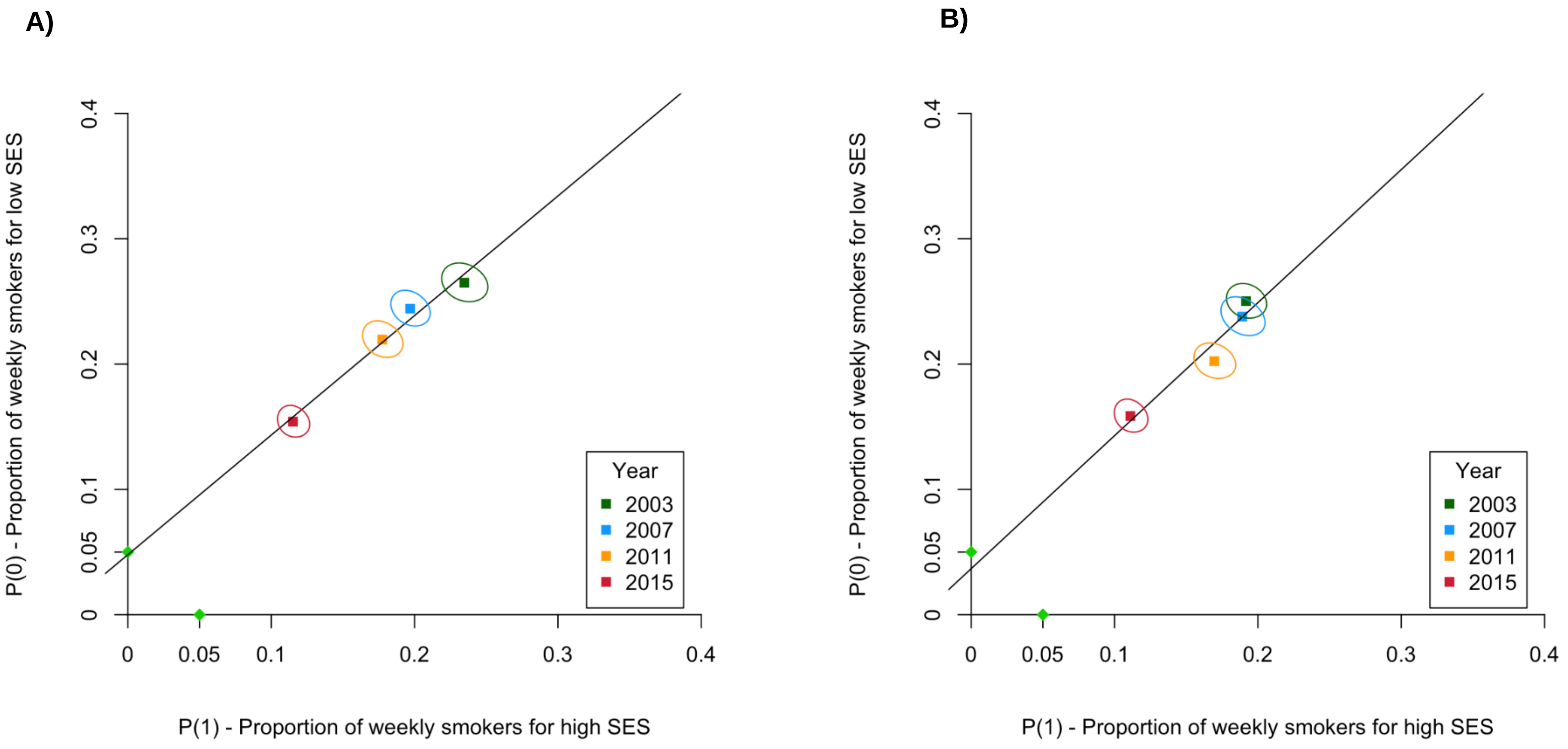

Web Figure 5: Linear trend in socioeconomic inequalities in smoking in 23 European countries among boys (**A.**) and girls (**B.**), with fitted regression line and 95% confidence regions for each time point. (Results derived from weighted and imputed data) Data from 23 European countries of the ESPAD study in four survey waves (2003- 2015).

Web Appendix 1: Details regarding the statistical methods used for each step of the proposed methodology.

1. Estimation of smoking prevalence at both ends of the SES scale

Multilevel logistic regression models were run using the *melogit* command in Stata v.15.0. For all analyses the variable cluster was used as the random effect, weekly smoking status was the dependent variable, and SES was the independent variable. From each of these models, we obtained $\hat{b}_0$, $\hat{b}_1$, $\widehat{\mathrm{Var}}(\hat{b}_0)$, $\widehat{\mathrm{Var}}(\hat{b}_1)$, and $\widehat{\mathrm{Cov}}(\hat{b}_0, \hat{b}_1)$. Table S1 presents the estimated parameters and the estimated elements of the variance-covariance matrix for these models. We used a robust estimator for the variance-covariance matrix (using the command vce(robust)). The interpretation of each estimate is as follows:

$\hat{b}_0$: the estimated intercept, i.e., the estimated log(odds) of smoking in the low SES group (SES=0)
$\hat{b}_1$: the estimated SES coefficient, i.e., the estimated additional log(odds) of smoking for the high SES group (SES=1) compared with the low SES group (SES=0)
$\widehat{\mathrm{Var}}(\hat{b}_0)$: estimated variance of the estimated intercept
$\widehat{\mathrm{Var}}(\hat{b}_1)$: estimated variance of the estimated SES coefficient
$\widehat{\mathrm{Cov}}(\hat{b}_0, \hat{b}_1)$: estimated covariance of the estimated intercept and the estimated SES coefficient

We calculated estimates for the true proportion of weekly smokers among low SES adolescents (SES=0), referred to as $P(0)$, and among high SES adolescents (SES=1), referred to as $P(1)$, by using the estimated intercept and estimated SES coefficient from each regression model. Specifically, we plugged in each pair of values of $\hat{b}_0$ and $\hat{b}_1$ in the formulas for $P(0)$ and $P(1)$ listed below, separately for each survey year and gender:

$$P(0) = \frac{e^{\hat{b}_0}}{1 + e^{\hat{b}_0}}, \qquad P(1) = \frac{e^{\hat{b}_0 + \hat{b}_1}}{1 + e^{\hat{b}_0 + \hat{b}_1}}$$

We constructed 95% confidence intervals (95% CIs) for the true proportions of weekly smokers which are estimated by $P(0)$ and $P(1)$, respectively. We used a simulation-based approach to construct these 95% CIs. Although these 95% CIs could be constructed by using alternative methods (e.g., the Delta method), the calculation of the likelihood of eradication from step 5 requires the use of simulations, and these simulations easily provide these 95% CIs as a by-product. We simulated 10,000 values for $\hat{b}_0$ and $\hat{b}_1$ from the bivariate normal distribution (separately for each survey year and gender):

$$N\left(\begin{pmatrix}\hat{b}_0\\ \hat{b}_1\end{pmatrix}, \begin{pmatrix}\widehat{\mathrm{Var}}(\hat{b}_0) & \widehat{\mathrm{Cov}}(\hat{b}_0, \hat{b}_1)\\ \widehat{\mathrm{Cov}}(\hat{b}_0, \hat{b}_1) & \widehat{\mathrm{Var}}(\hat{b}_1)\end{pmatrix}\right)$$

These 10,000 simulated values for $\hat{b}_0$ and $\hat{b}_1$ were used to generate 10,000 simulated values for $P(0)$ and $P(1)$ using the above formulas. We then used the 2.5% and 97.5% percentiles of the distributions of the 10,000 simulated values for $P(0)$ and $P(1)$, respectively, to create the corresponding 95% CIs. Table S2 presents the values of $P(0)$ and $P(1)$ with corresponding 95% CIs, separately for each survey year and gender.

2. Prevalence points and 95% confidence regions (95% CRs)

We plotted the values of $P(0)$ and $P(1)$ against each other for each survey year. This resulted in a graph with four points: one point with coordinates ($P(1)$,$P(0)$) for each survey year. Separate graphs were created for boys and girls, and for all countries combined and by European region.

In the graphs, we wanted to describe the uncertainty around the prevalence points ($P(1)$,$P(0)$). The 10,000 simulated values for $\hat{b}_0$ and from step 1 were used to create corresponding ellipse-shaped 95% CRs for (b_0, b_1), separately for each survey year and gender. The ellipses that contain 95% of the simulated values were constructed using the function DataEllipse() from R. These 95% CRs were subsequently transformed to create 95% CRs that correspond to the prevalence points ($P(1)$,$P(0)$).

3. Fitting the regression line for the linear relationship between low and high SES prevalence

The four points with coordinates ($P(1)$,$P(0)$) were subsequently used to fit a linear regression line, which was presented in each graph. This regression line shows the linear relationship between low and high SES smoking prevalence over time. From this regression line we calculated the intercept (i.e., the point where the

regression line crosses the y-axis), which indicates the expected prevalence among low SES adolescents when the prevalence among high SES adolescents reached 0%.

4. 95% confidence interval for the intercept of the regression line

We used the simulated 10,000 values for $(P(1),P(0))$ from step 1 to fit 10,000 regression lines for the linear relationship between low and high SES smoking prevalence written compactly as: $P(0)_{ij} = \alpha_i + \beta_i P(1)_{ij} + \varepsilon_{ij}$, $i = 1\ to\ 10{,}000$ (10,000 simulations) and $j = 1\ to\ 4$ (4 time points). Each one of these regression lines had an intercept, and we used the 2.5% and 97.5% percentiles of the distribution of these 10,000 intercept values to constructed a 95% CI for the intercept of the linear relationship between low and high SES smoking prevalence. This 95% CI indicates the uncertainty around the value that the smoking prevalence among low SES adolescents will have when the prevalence among high SES adolescents is 0%.

5. Likelihood of eradication

In step 5 we used the 10,000 regression lines for the linear relationship between low and high SES smoking prevalence that were fitted in step 4. For each fitted regression line, we determined the intersection point with the axes; this may be the intercept if the y-axis is crossed first, or the point where the x-axis is crossed if the x-axis is crossed first. We calculated the likelihood of eradication as the percentage of the 10,000 fitted lines for which the intersection point fell within the desired scenario of <5% smoking prevalence among low and high SES adolescents (i.e., within 0.05 from the origin).

Web Appendix 2: Unweighted analyses in Stata

```
import delimited
"C:\Users\kangk\Box\Research\SES\data\data_with_cluster_2022Aug10.csv",
clear
* replace NA with "" (missing string)
replace ses = "" if ses == "NA"
replace smokweekly = "" if smokweekly == "NA"
* convert strings to numbers
destring ses smokweekly, replace

* All Regions

melogit smokweekly ses if year == 0 & sex== 1 ||cluster:, vce(robust)
matrix list e(V)


melogit smokweekly ses if year == 1 & sex== 1 ||cluster:, vce(robust)
matrix list e(V)


melogit smokweekly ses if year == 2 & sex== 1 ||cluster:, vce(robust)
matrix list e(V)


melogit smokweekly ses if year == 3 & sex== 1 ||cluster:, vce(robust)
matrix list e(V)


melogit smokweekly ses if year == 0 & sex== 0 ||cluster:, vce(robust)
matrix list e(V)


melogit smokweekly ses if year == 1 & sex== 0 ||cluster:, vce(robust)
matrix list e(V)


melogit smokweekly ses if year == 2 & sex== 0 ||cluster:, vce(robust)
matrix list e(V)


melogit smokweekly ses if year == 3 & sex== 0 ||cluster:, vce(robust)
matrix list e(V)


* Region 0
melogit smokweekly ses if year == 0 & sex== 1 & region == 0 ||cluster:,
vce(robust)
matrix list e(V)

melogit smokweekly ses if year == 1 & sex== 1 & region == 0 ||cluster:,
vce(robust)
matrix list e(V)

melogit smokweekly ses if year == 2 & sex== 1 & region == 0 ||cluster:,
vce(robust)
matrix list e(V)

melogit smokweekly ses if year == 3 & sex== 1 & region == 0 ||cluster:,
vce(robust)
matrix list e(V)

melogit smokweekly ses if year == 0 & sex== 0 & region == 0 ||cluster:,
vce(robust)
matrix list e(V)

melogit smokweekly ses if year == 1 & sex== 0 & region == 0 ||cluster:,
vce(robust)
matrix list e(V)
```

```
melogit smokweekly ses if year == 2 & sex== 0 & region == 0 ||cluster:,
vce(robust)
matrix list e(V)

melogit smokweekly ses if year == 3 & sex== 0 & region == 0 ||cluster:,
vce(robust)
matrix list e(V)

*Region 1
melogit smokweekly ses if year == 0 & sex== 1 & region == 1 ||cluster:,
vce(robust)
matrix list e(V)

melogit smokweekly ses if year == 0 & sex== 1 & region == 1 ||cluster:,
vce(robust)
matrix list e(V)

melogit smokweekly ses if year == 1 & sex== 1 & region == 1 ||cluster:,
vce(robust)
matrix list e(V)

melogit smokweekly ses if year == 2 & sex== 1 & region == 1 ||cluster:,
vce(robust)
matrix list e(V)

melogit smokweekly ses if year == 3 & sex== 1 & region == 1 ||cluster:,
vce(robust)
matrix list e(V)

melogit smokweekly ses if year == 0 & sex== 0 & region == 1 ||cluster:,
vce(robust)
matrix list e(V)

melogit smokweekly ses if year == 1 & sex== 0 & region == 1 ||cluster:,
vce(robust)
matrix list e(V)

melogit smokweekly ses if year == 2 & sex== 0 & region == 1 ||cluster:,
vce(robust)
matrix list e(V)

melogit smokweekly ses if year == 3 & sex== 0 & region == 1 ||cluster:,
vce(robust)
matrix list e(V)

*Region 2
melogit smokweekly ses if year == 0 & sex== 1 & region == 2 ||cluster:,
vce(robust)
matrix list e(V)

melogit smokweekly ses if year == 1 & sex== 1 & region == 2 ||cluster:,
vce(robust)
matrix list e(V)

melogit smokweekly ses if year == 2 & sex== 1 & region == 2 ||cluster:,
vce(robust)
matrix list e(V)

melogit smokweekly ses if year == 3 & sex== 1 & region == 2 ||cluster:,
vce(robust)
matrix list e(V)

melogit smokweekly ses if year == 0 & sex== 0 & region == 2 ||cluster:,
vce(robust)
matrix list e(V)

melogit smokweekly ses if year == 1 & sex== 0 & region == 2 ||cluster:,
vce(robust)
matrix list e(V)
```

```
melogit smokweekly ses if year == 2 & sex== 0 & region == 2 ||cluster:,
vce(robust)
matrix list e(V)

melogit smokweekly ses if year == 3 & sex== 0 & region == 2 ||cluster:,
vce(robust)
matrix list e(V)

*Region 3
melogit smokweekly ses if year == 0 & sex== 1 & region == 3 ||cluster:,
vce(robust)
matrix list e(V)

melogit smokweekly ses if year == 1 & sex== 1 & region == 3 ||cluster:,
vce(robust)
matrix list e(V)

melogit smokweekly ses if year == 2 & sex== 1 & region == 3 ||cluster:,
vce(robust)
matrix list e(V)

melogit smokweekly ses if year == 3 & sex== 1 & region == 3 ||cluster:,
vce(robust)
matrix list e(V)

melogit smokweekly ses if year == 0 & sex== 0 & region == 3 ||cluster:,
vce(robust)
matrix list e(V)

melogit smokweekly ses if year == 1 & sex== 0 & region == 3 ||cluster:,
vce(robust)
matrix list e(V)

melogit smokweekly ses if year == 2 & sex== 0 & region == 3 ||cluster:,
vce(robust)
matrix list e(V)

melogit smokweekly ses if year == 3 & sex== 0 & region == 3 ||cluster:,
vce(robust)
matrix list e(V)
```

Web Appendix 3: Weighted analysis in Stata

```
import delimited
"C:\Users\kangk\Box\Research\SES\new_analysis_Aug3\ESPAD_inputation_correct
ed.csv", clear

* All Regions
melogit smokweekly ses if year == 0 & sex== 1 [pweight= weights
]||cluster:, vce(robust)
matrix list e(V)


melogit smokweekly ses if year == 1 & sex== 1 [pweight= weights
]||cluster:, vce(robust)
matrix list e(V)


melogit smokweekly ses if year == 2 & sex== 1 [pweight= weights
]||cluster:, vce(robust)
matrix list e(V)


melogit smokweekly ses if year == 3 & sex== 1 [pweight= weights
]||cluster:, vce(robust)
matrix list e(V)


melogit smokweekly ses if year == 0 & sex== 0 [pweight= weights
]||cluster:, vce(robust)
matrix list e(V)


melogit smokweekly ses if year == 1 & sex== 0 [pweight= weights
]||cluster:, vce(robust)
matrix list e(V)


melogit smokweekly ses if year == 2 & sex== 0 [pweight= weights
]||cluster:, vce(robust)
matrix list e(V)


melogit smokweekly ses if year == 3 & sex== 0 [pweight= weights
]||cluster:, vce(robust)
matrix list e(V)


* Region 0
melogit smokweekly ses if year == 0 & sex== 1 & region == 0 [pweight=
weights ]||cluster:, vce(robust)
matrix list e(V)

melogit smokweekly ses if year == 1 & sex== 1 & region == 0 [pweight=
weights ]||cluster:, vce(robust)
matrix list e(V)

melogit smokweekly ses if year == 2 & sex== 1 & region == 0 [pweight=
weights ]||cluster:, vce(robust)
matrix list e(V)

melogit smokweekly ses if year == 3 & sex== 1 & region == 0 [pweight=
weights ]||cluster:, vce(robust)
matrix list e(V)

melogit smokweekly ses if year == 0 & sex== 0 & region == 0 [pweight=
weights ]||cluster:, vce(robust)
matrix list e(V)
```

```
melogit smokweekly ses if year == 1 & sex== 0 & region == 0 [pweight=
weights ]||cluster:, vce(robust)
matrix list e(V)

melogit smokweekly ses if year == 2 & sex== 0 & region == 0 [pweight=
weights ]||cluster:, vce(robust)
matrix list e(V)

melogit smokweekly ses if year == 3 & sex== 0 & region == 0 [pweight=
weights ]||cluster:, vce(robust)
matrix list e(V)

*Region 1
melogit smokweekly ses if year == 0 & sex== 1 & region == 1 [pweight=
weights ]||cluster:, vce(robust)
matrix list e(V)

melogit smokweekly ses if year == 1 & sex== 1 & region == 1 [pweight=
weights ]||cluster:, vce(robust)
matrix list e(V)

melogit smokweekly ses if year == 2 & sex== 1 & region == 1 [pweight=
weights ]||cluster:, vce(robust)
matrix list e(V)

melogit smokweekly ses if year == 3 & sex== 1 & region == 1 [pweight=
weights ]||cluster:, vce(robust)
matrix list e(V)

melogit smokweekly ses if year == 0 & sex== 0 & region == 1 [pweight=
weights ]||cluster:, vce(robust)
matrix list e(V)

melogit smokweekly ses if year == 1 & sex== 0 & region == 1 [pweight=
weights ]||cluster:, vce(robust)
matrix list e(V)

melogit smokweekly ses if year == 2 & sex== 0 & region == 1 [pweight=
weights ]||cluster:, vce(robust)
matrix list e(V)

melogit smokweekly ses if year == 3 & sex== 0 & region == 1 [pweight=
weights ]||cluster:, vce(robust)
matrix list e(V)

*Region 2
melogit smokweekly ses if year == 0 & sex== 1 & region == 2 [pweight=
weights ]||cluster:, vce(robust)
matrix list e(V)

melogit smokweekly ses if year == 1 & sex== 1 & region == 2 [pweight=
weights ]||cluster:, vce(robust)
matrix list e(V)

melogit smokweekly ses if year == 2 & sex== 1 & region == 2 [pweight=
weights ]||cluster:, vce(robust)
matrix list e(V)

melogit smokweekly ses if year == 3 & sex== 1 & region == 2 [pweight=
weights ]||cluster:, vce(robust)
matrix list e(V)

melogit smokweekly ses if year == 0 & sex== 0 & region == 2 [pweight=
weights ]||cluster:, vce(robust)
matrix list e(V)

melogit smokweekly ses if year == 1 & sex== 0 & region == 2 [pweight=
weights ]||cluster:, vce(robust)
matrix list e(V)
```

```
melogit smokweekly ses if year == 2 & sex== 0 & region == 2 [pweight=
weights ]||cluster:, vce(robust)
matrix list e(V)

melogit smokweekly ses if year == 3 & sex== 0 & region == 2 [pweight=
weights ]||cluster:, vce(robust)
matrix list e(V)

*Region 3
melogit smokweekly ses if year == 0 & sex== 1 & region == 3 [pweight=
weights ]||cluster:, vce(robust)
matrix list e(V)

melogit smokweekly ses if year == 1 & sex== 1 & region == 3 [pweight=
weights ]||cluster:, vce(robust)
matrix list e(V)

melogit smokweekly ses if year == 2 & sex== 1 & region == 3 [pweight=
weights ]||cluster:, vce(robust)
matrix list e(V)

melogit smokweekly ses if year == 3 & sex== 1 & region == 3 [pweight=
weights ]||cluster:, vce(robust)
matrix list e(V)

melogit smokweekly ses if year == 0 & sex== 0 & region == 3 [pweight=
weights ]||cluster:, vce(robust)
matrix list e(V)

melogit smokweekly ses if year == 1 & sex== 0 & region == 3 [pweight=
weights ]||cluster:, vce(robust)
matrix list e(V)

melogit smokweekly ses if year == 2 & sex== 0 & region == 3 [pweight=
weights ]||cluster:, vce(robust)
matrix list e(V)

melogit smokweekly ses if year == 3 & sex== 0 & region == 3 [pweight=
weights ]||cluster:, vce(robust)
matrix list e(V)
```

Web Appendix 4: R code

```
## ---- message = FALSE, warning = FALSE------------------------------------
-------------------------------------------------
library(car)
library(table1)
library(magrittr)
library(rms)
library(kableExtra)


## -------------------------------------------------------------------------
-------------------------------------------------
# Unweighted Data
data = read.csv("ESPAD_base_for_inputation.csv", header = TRUE)

## -------------------------------------------------------------------------
-------------------------------------------------
data$year_lab = data$year
data$year_lab %<>% factor(labels = c("2003", "2007", "2011", "2015"))
data$year %<>% factor()
data$sex_lab = data$sex
data$sex_lab %<>% factor(labels = c("Girls", "Boys"))
data$sex %<>% factor()
# data$region %<>% factor(labels = c("North", "East", "South", "West"))

## -------------------------------------------------------------------------
-------------------------------------------------
est <- read.csv("stata_estimates_unweighted.csv", header=TRUE,
fileEncoding="UTF-8-BOM")

## -------------------------------------------------------------------------
-------------------------------------------------

######### definition of plot-producing function ########
# data: original data set
# est: the data frame containing the estimates from STATA

produce.plot <- function( data=data, est=est, region, gdr, rep, x_limit,
y_limit){

  if (region == 99) {
    sub.data = data
  } else {
    sub.data = data[data$region == region & data$sex == gdr,]
  }

  sub.est = est[est$region == region & est$sex == gdr,]

  #sample size
  sample.size = NULL
  for (r in 1:nrow(sub.est)){
    sample.size[r] = sum(sub.data$sex == sub.data$sex[r] & sub.data$year ==
sub.data$year[r])
  }
  est = cbind(sub.est, sample.size)

  s_p0 = array(NA, dim = c(nrow(sub.est), rep))
  s_p1 = array(NA, dim = c(nrow(sub.est), rep))
  sv = list()


  ## draw the confidence regions/ellipses

  # year 0
  k0 = sub.est$sex==gdr & sub.est$year == 0
  mu0 = c(sub.est$cons[k0], sub.est$b_ses[k0])
  cov0 = matrix(c(sub.est$var_cons[k0], sub.est$covar[k0],
sub.est$covar[k0], sub.est$var_ses[k0]), nrow = 2)
```

```
  y0 = mvtnorm::rmvnorm(n=rep, mean = mu0, sigma=cov0)

  # year 1
  k1 = sub.est$sex==gdr & sub.est$year == 1
  mu1 = c(sub.est$cons[k1], sub.est$b_ses[k1])
  cov1 = matrix(c(sub.est$var_cons[k1], sub.est$covar[k1],
sub.est$covar[k1], sub.est$var_ses[k1]), nrow = 2)
  y1 = mvtnorm::rmvnorm(n=rep, mean = mu1, sigma=cov1)

  # year 2
  k2 = sub.est$sex==gdr & sub.est$year == 2
  mu2 = c(sub.est$cons[k2], sub.est$b_ses[k2])
  cov2 = matrix(c(sub.est$var_cons[k2], sub.est$covar[k2],
sub.est$covar[k2], sub.est$var_ses[k2]), nrow = 2)
  y2 = mvtnorm::rmvnorm(n=rep, mean = mu2, sigma=cov2)

  # year 3
  k3 = sub.est$sex==gdr & sub.est$year == 3
  mu3 = c(sub.est$cons[k3], sub.est$b_ses[k3])
  cov3 = matrix(c(sub.est$var_cons[k3], sub.est$covar[k3],
sub.est$covar[k3], sub.est$var_ses[k3]), nrow = 2)
  y3 = mvtnorm::rmvnorm(n=rep, mean = mu3, sigma=cov3)


  # the points on the ellipses
  E0 = dataEllipse( y0,levels = 0.95, center.pch = 5, plot.points = FALSE,
add = TRUE, fill = TRUE, lwd = 1, col = "blue", draw = FALSE)

  E1 = dataEllipse( y1,levels = 0.95, center.pch = 5, plot.points = FALSE,
add = TRUE, fill = TRUE, lwd = 1, col = "blue", draw = FALSE)

  E2 = dataEllipse( y2,levels = 0.95, center.pch = 5, plot.points = FALSE,
add = TRUE, fill = TRUE, lwd = 1, col = "blue", draw = FALSE)

  E3 = dataEllipse( y3,levels = 0.95, center.pch = 5, plot.points = FALSE,
add = TRUE, fill = TRUE, lwd = 1, col = "blue", draw = FALSE)

  # transformation of the ellipse
  t_E0 = cbind(
    exp(E0[,1]+E0[,2])/(1+exp(E0[,1]+E0[,2])), exp(E0[,1])/(1+exp(E0[,1]))
  )

  t_E1 = cbind(
    exp(E1[,1]+E1[,2])/(1+exp(E1[,1]+E1[,2])), exp(E1[,1])/(1+exp(E1[,1]))
  )

  t_E2 = cbind(
    exp(E2[,1]+E2[,2])/(1+exp(E2[,1]+E2[,2])), exp(E2[,1])/(1+exp(E2[,1]))
  )

  t_E3 = cbind(
    exp(E3[,1]+E3[,2])/(1+exp(E3[,1]+E3[,2])), exp(E3[,1])/(1+exp(E3[,1]))
  )

  # the mean p0 and p1 for each year and gender
  p0_mu = exp(sub.est$cons)/(1+exp(sub.est$cons))
  p1_mu = exp(sub.est$cons + sub.est$b_ses)/(1 + exp(sub.est$cons +
sub.est$b_ses))

  # tranform to obtain the simulated P(0) and P(1)
  ## P(0)
  s_p0[1, ] = exp(y0[ , 1])/(1 + exp(y0[ , 1])) # year 0
  s_p0[2, ] = exp(y1[ , 1])/(1 + exp(y1[ , 1])) # year 1
  s_p0[3, ] = exp(y2[ , 1])/(1 + exp(y2[ , 1])) # year 2
  s_p0[4, ] = exp(y3[ , 1])/(1 + exp(y3[ , 1])) # year 3

  ## P(1)
  s_p1[1, ] = exp(y0[ , 1] + y0[, 2]) / (1 + exp(y0[ , 1] + y0[, 2]) ) #
year 0
```

```
  s_p1[2, ] = exp(y1[ , 1] + y1[, 2]) / (1 + exp(y1[ , 1] + y1[, 2]) ) #
year 1
  s_p1[3, ] = exp(y2[ , 1] + y2[, 2]) / (1 + exp(y2[ , 1] + y2[, 2]) ) #
year 2
  s_p1[4, ] = exp(y3[ , 1] + y3[, 2]) / (1 + exp(y3[ , 1] + y3[, 2]) ) #
year 3


  # combining
  sp0.value = cbind(sub.est[, 1:3], p0_mu, s_p0)
  sp1.value = cbind(sub.est[, 1:3], p1_mu, s_p1)

  # simulation of the trends

  alpha = NULL # recording the estimated intercepts

  slope = NULL # recording the beta's

  points = matrix(NA, ncol=2, nrow=rep) # record the crossing points

  area = matrix(NA, ncol=4, nrow=rep) # record the dummy variables
indicating the areas

  for (j in 1:rep){

    y = sp0.value[sp0.value$sex == gdr, j+4] # y-axis -> P(0)

    x = sp1.value[sp0.value$sex == gdr, j+4] # x-axis -> P(1)

    model = lm(y~x)

    alpha[j] = coef(model)[1]

    slope[j] = coef(model)[2]

    # the crossing points of each line that across the axis

    if (alpha[j] >= 0) points[j, ] = c(0, alpha[j])

    if (alpha[j] < 0) points[j, ] = c(-alpha[j]/slope[j],0)

    # judge the area of the crossing points
    if (points[j, 1] == 0 & points[j, 2] >= 0.05) area[j, ] = c(1, 0, 0, 0)
    if (points[j, 1] == 0 & points[j, 2] < 0.05 & 0 < points[j, 2]) area[j,
] = c(0, 1, 0, 0)
    if (0 < points[j, 1] & points[j, 1] < 0.05 & points[j, 2] == 0) area[j,
] = c(0, 0, 1, 0)
    if (points[j, 1] >= 0.05 & points[j, 2] == 0) area[j, ] = c(0, 0, 0, 1)

  } #loop j

  # 95% CI for the estimates of intercepts
  CI_alpha = quantile(alpha, probs = c(0.025, 0.975))

  # obtain the area probability distribution of crossing points

  prob = apply(area, 2, mean)

  ############################################################
  # producing plots
  ############################################################
  options(digits = 5)

  ## Main plot
  value0 = rbind(sp1.value$p1_mu[sp1.value$sex == gdr],
                 sp0.value$p0_mu[sp1.value$sex == gdr])
  value0 = t(value0)
  #column1-> P1
  #column2-> P0
```

```
  plot(value0,
       xlim = x_limit, ylim = y_limit ,
       pch =15, col=c("#006600", "#0099FF", "#FF9900", "#CC0033"),
       #xaxs = "i", yaxs = "i",
       xlab="P(1) - Proportion of weekly smokers for high SES", ylab="P(0)
- Proportion of weekly smokers for low SES",
       # main  = paste(ctry.name$country.name[ctry.name$code == ctry], "-",
gdr.label$gender.label[gdr.label$code == gdr], sep = ""),
       bty = "l",
       axes = FALSE
  )
  axis(2, pos = 0, at = c(0, 0.05, 0.1, 0.2, 0.3, 0.4),
       labels = c('0', '0.05', '0.1', '0.2', '0.3', '0.4')
  )
  axis(1, pos = 0, at = c(0, 0.05, 0.1, 0.2, 0.3, 0.4),
       labels = c('0', '0.05', '0.1', '0.2', '0.3', '0.4')
       )

  #text(value0, labels = c("2003","2007", "2011", "2015"), pos = position)
  legend(0.32, 0.13, pch = 15, title = c('Year'),
         c("2003","2007", "2011", "2015"),
         col = c("#006600", "#0099FF", "#FF9900", "#CC0033"),
         )

  #abline(v=0,h=0)
  #abline(v=0.05, h=0.05, lty=2)
  # No dotted lines, indicators instead

  points(0.05, 0, pch = 18, col = "green3", lwd = 2, cex = 1.3)
  points(0, 0.05, pch = 18, col = "green3", lwd = 2, cex = 1.3)

  p1 = value0[, 1] # x-axis
  p0 = value0[, 2] # y-axis

  model0 = lm(p0~p1)
  abline(model0, lwd=1)

  # Draw the ellipses
  points(t_E0, type="l", col = "#006600")
  #polygon(t_E0, col=rgb(3)

  points(t_E1, type="l", col = "#0099FF")
  #polygon(t_E1, col=4)

  points(t_E2, type="l", col = "#FF9900")
  #polygon(t_E2, col=5)

  points(t_E3, type="l", col = "#CC0033")
  #polygon(t_E3, col=6)

  # Compute separate CIs for P(0) and P(1) based on quantiles
  if (gdr == 1) {
    sp0.value.gender = sp0.value[sp0.value$sex == 1,]
    sp1.value.gender = sp1.value[sp1.value$sex == 1,]
  }
  if (gdr == 0) {
    sp0.value.gender = sp0.value[sp0.value$sex == 0,]
    sp1.value.gender = sp1.value[sp1.value$sex == 0,]
  }

  LL2_P0 = unlist(c(quantile(sp0.value.gender[1, ], probs = 0.025),
quantile(sp0.value.gender[2, ], probs = 0.025),
                    quantile(sp0.value.gender[3, ], probs = 0.025),
quantile(sp0.value.gender[4, ], probs = 0.025)))
  UL2_P0 = unlist(c(quantile(sp0.value.gender[1, ], probs = 0.975),
quantile(sp0.value.gender[2, ], probs = 0.975),
                    quantile(sp0.value.gender[3, ], probs = 0.975),
quantile(sp0.value.gender[4, ], probs = 0.975)))
```

```
  LL2_P1 = unlist(c(quantile(sp1.value.gender[1, ], probs = 0.025),
quantile(sp1.value.gender[2, ], probs = 0.025),
                    quantile(sp1.value.gender[3, ], probs = 0.025),
quantile(sp1.value.gender[4, ], probs = 0.025)))
  UL2_P1 = unlist(c(quantile(sp1.value.gender[1, ], probs = 0.975),
quantile(sp1.value.gender[2, ], probs = 0.975),
                    quantile(sp1.value.gender[3, ], probs = 0.975),
quantile(sp1.value.gender[4, ], probs = 0.975)))

  temp.table = data.frame(region = c(region, NA, NA, NA),
                          intercept = c(model0$coefficients[1], NA, NA,
NA),
                          LL_intercept = c(CI_alpha[1], NA, NA, NA),
                          UL_intercept = c(CI_alpha[2], NA, NA, NA),
                          slope = c(model0$coefficients[2], NA, NA, NA),
                          area_1 = c(prob[1], NA, NA, NA),
                          area_2 = c(prob[2], NA, NA, NA),
                          area_3 = c(prob[3], NA, NA, NA),
                          area_4 = c(prob[4], NA, NA, NA),
                          Year = c(2003, 2007, 2011, 2015),
                          P0_hat = p0_mu[sub.est$sex==gdr],
                          LL_P0 = c(min(t_E0[,2]), min(t_E1[,2]),
min(t_E2[,2]), min(t_E3[,2])),
                          UL_P0 = c(max(t_E0[,2]), max(t_E1[,2]),
max(t_E2[,2]), max(t_E3[,2])),
                          LL2_P0 = LL2_P0,
                          UL2_P0 = UL2_P0,
                          P1_hat = p1_mu[sub.est$sex==gdr],
                          LL_P1 = c(min(t_E0[,1]), min(t_E1[,1]),
min(t_E2[,1]), min(t_E3[,1])),
                          UL_P1 = c(max(t_E0[,1]), max(t_E1[,1]),
max(t_E2[,1]), max(t_E3[,1])),
                          LL2_P1 = LL2_P1,
                          UL2_P1 = UL2_P1

  )


  return(temp.table)

} #end of function "produce.plot".


## ---------------------------------------------------------------------------
------------------------------------------------
# seed number
set.seed (0823) # to guarantee the repeatablity (randomly chosen)

rep = 10000  # num of simulations.

x_limit = c(0, 0.4)  # the ranges of x- and y-axes on figures
y_limit = c(0, 0.4)

# position = matrix(
#   c(
#     1, 3, 1, 3
#     ),   #1:down; 2:left; 3:up; 4:right
#   ncol = 4, byrow = TRUE
# )

ctry.name = list(code = c( 99),
                country.name = c("All countries"))

gdr.label = list(code = 0:1,
                    gender.label = c("Female", "Male")
                    )


## ---- fig.width=6, fig.height=6----------------------------------------
------------------------------------------------
```

```
produce.plot(data=data, est=est, region = 99, gdr = 1, rep = rep,
x_limit=x_limit, y_limit=y_limit)
title("All Regions - Boys")


## ---- fig.width=6, fig.height=6------------------------------------------
-----------------------------------------------
produce.plot(data=data, est=est, region = 99, gdr = 0, rep = rep,
x_limit=x_limit, y_limit=y_limit)
title("All Regions - Girls")


## ---- fig.width=6, fig.height=6------------------------------------------
-----------------------------------------------
produce.plot(data=data, est=est, region = 0, gdr = 1, rep = rep,
x_limit=x_limit, y_limit=y_limit)
title("Region 0 (North) - Boys")


## ---- fig.width=6, fig.height=6------------------------------------------
-----------------------------------------------
produce.plot(data=data, est=est, region = 0, gdr = 0, rep = rep,
x_limit=x_limit, y_limit=y_limit)
title("Region 0 (North) - Girls")


## ---- fig.width=6, fig.height=6------------------------------------------
-----------------------------------------------
produce.plot(data=data, est=est, region = 1, gdr = 1, rep = rep,
x_limit=x_limit, y_limit=y_limit)
title("Region 1 (East) - Boys")


## ---- fig.width=6, fig.height=6------------------------------------------
-----------------------------------------------
produce.plot(data=data, est=est, region = 1, gdr = 0, rep = rep,
x_limit=x_limit, y_limit=y_limit)
title("Region 1 (East) - Girls")


## ---- fig.width=6, fig.height=6------------------------------------------
-----------------------------------------------
produce.plot(data=data, est=est, region = 2, gdr = 1, rep = rep,
x_limit=x_limit, y_limit=y_limit)
title("Region 2 (South) - Boys")


## ---- fig.width=6, fig.height=6------------------------------------------
-----------------------------------------------
produce.plot(data=data, est=est, region = 2, gdr = 0, rep = rep,
x_limit=x_limit, y_limit=y_limit)
title("Region 2 (South) - Girls")


## ---- fig.width=6, fig.height=6------------------------------------------
-----------------------------------------------
produce.plot(data=data, est=est, region = 3, gdr = 1, rep = rep,
x_limit=x_limit, y_limit=y_limit)
title("Region 3 (West) - Boys")


## ---- fig.width=6, fig.height=6------------------------------------------
-----------------------------------------------
produce.plot(data=data, est=est, region = 3, gdr = 0, rep = rep,
x_limit=x_limit, y_limit=y_limit)
title("Region 3 (West) - Girls")
```
